# Dealing with Consumer Differences in Liking during Repeated Exposure to Food; Typical Dynamics in Rating Behavior

**DOI:** 10.1371/journal.pone.0093350

**Published:** 2014-03-25

**Authors:** Jelle R. Dalenberg, Luca Nanetti, Remco J. Renken, René A. de Wijk, Gert J. ter Horst

**Affiliations:** 1 Top Institute Food and Nutrition, Wageningen, the Netherlands; 2 Neuroimaging Center Groningen, University Medical Center Groningen, Groningen, The Netherlands; 3 Centre for Innovative Consumer Studies, Wageningen University and Research Centre, Wageningen, the Netherlands; University of Missouri-Kansas City, United States of America

## Abstract

Consumers show high interindividual variability in food liking during repeated exposure. To investigate consumer liking during repeated exposure, data is often interpreted on a product level by averaging results over all consumers. However, a single product may elicit inconsistent behaviors in consumers; averaging will mix and hide possible subgroups of consumer behaviors, leading to a misinterpretation of the results. To deal with the variability in consumer liking, we propose to use clustering on data from consumer-product combinations to investigate the nature of the behavioral differences within the complete dataset. The resulting behavioral clusters can then be used to describe product acceptance. To test this approach we used two independent data sets in which young adults were repeatedly exposed to drinks and snacks, respectively. We found that five typical consumer behaviors existed in both datasets. These behaviors differed both in the average level of liking as well as its temporal dynamics. By investigating the distribution of a single product across typical consumer behaviors, we provide more precise insight in how consumers divide in subgroups based on their product liking (i.e. product modality). This work shows that taking into account and using interindividual differences can unveil information about product acceptance that would otherwise be ignored.

## Introduction

Consumers show high variability in product liking. For a single product, differences in liking both develop between and within consumers during multiple exposures over time [1]–[10]. Three types of liking behaviors are typically found during repeated exposure: a decrease in liking, an increase in liking and no significant changes in liking. Several studies have shown that a product can become more liked in the context of repeated exposure in both children and adults [2]–[5]. Mere repeated exposure is known to induce an increase in liking [11], an effect that Pliner [2] associated with the dissipation of neophobia. However, this effect is mostly reported for (novel) products that received an initial negative or neutral hedonic value. For products that are initially rated as pleasant, repeated exposure does not necessarily increase or stabilize liking. Multiple studies showed that liking for initially liked products such as chocolate, soups and juices, can decrease during multiple exposures over time [6], [7], [9], [12], [13]. This negative effect is associated with product boredom: after multiple exposures a consumer becomes bored with that specific food stimulus. Intriguingly, Weijzen et al. [12] could not attribute a decline in liking to boredom, arguing that a decline in liking may rather reflect a more general loss in interest in the food. The effect of boredom (or loss of interest) can partially be countered by the offered stimulus range: when more variety is offered during repeated exposure, consumers report lower boredom [14]. Consumers can also show stable liking for products. Stable liking was found in groups that repeatedly consumed certain products or combinations thereof, such as for instance bread and butter, or certain types of soups and juices [7], [9], [13], [15].

To the best of our knowledge previous studies have mostly investigated the effects of repeated exposure on a product level (i.e. averaging the data of all participants per product). Averaging, however, hides interindividual differences and does not enable the researcher to investigate the nature of the differences between subject behaviors. Köster [16] termed the problem of group averaging: ‘The fallacy of consumer uniformity’: the not necessarily true assumption that consumers behave similarly. This suggests that averaging would mix and hide possible subgroups of consumer behaviors.

Köster [16], indeed, argues that products can be uni-, bi- or multimodal. Here, product modality refers to how consumers are divided in subgroups based on their product liking on a single product. Unimodal products are products that are similarly rated by all consumers, whereas bimodel products refer to products that are differently rated by two main groups, (e.g. likers and dislikers) and multimodal products are products that show three or more consumer subgroups in liking.

Recently, Kremer et al. [10] and Hoek et al. [9] addressed the fallacy of consumer uniformity by focusing on the liking slopes of every single participant-product combination. The authors reasoned that slopes for patterns related to mere repeated exposure, boredom/loss of interest and stable behavior, have positive, negative or non-significant slopes, respectively. The drawback of this approach is that information about the slope only informs about pattern changes, while liking cannot be discriminated from disliking when the intercept is ignored.

We propose to extend previous work by clustering the temporal liking behaviors occurring both within and between participants over all products under investigation. This shifts the focus from products and/or consumers to consumer-product combinations and allows using the complete dataset to explore the behavioral patterns in the consumer group. A proof of the principle for this approach was given by Moskowitz et al [17]. The authors demonstrated that clustering liking ratings given on a range of pasta sauces with increasing levels of spiciness successfully captured three behavioral subgroups; spice lovers, spice dislikers and optimum seekers. These results indicated that specific subgroups of consumers show unique liking behavior.

To find specific liking behaviors within a repeated exposure context, we applied the clustering approach on a repeated exposure study focused on commercially available drinks. For this study, we recruited participants to repeatedly taste and rate these products for 6 consecutive days.

To improve the ecological validity of the study, we included a wide range of complex taste stimuli that ranged from generally disliked to generally liked (regular off the shelf drinking products and Oral Nutritional Supplements (ONS)). Furthermore, intake of test stimuli was *ad libitum* rather than restricted.

In order to confirm our outcomes and to show generalizability, we repeated this analysis on a completely independent data set acquired by Weijzen et al. [12]. These authors set up a repeated exposure study in which participants were repeatedly exposed to a candy bar, chocolate and two types of biscuits for 5 days.

By applying the clustering technique to two independent data sets, we will show similarities in consumer behavior during repeated exposure to drinks and snacks. Furthermore, we will show that clustering reveals characteristic temporal liking patterns, which effectively identify typical consumer behaviors and provide insight in product modality.

## Method

### Study 1: Drinks

#### Participants

A total of 45 male Caucasian university students were recruited for two repeated exposure experiments. The participants were randomly assigned to two product-tasting groups. The first group (n = 22, μ_age_ = 24.67, σ_age_ = 3.37, range: 21–33) was recruited to taste ONS products in afternoon sessions on six consecutive days. The second group (n = 23, μ_age_ = 23.43, σ_age_ = 2.33, range: 21–28) was recruited to taste commercially available drinks during morning sessions on six consecutive days.

Time of the day was kept constant within the groups, because the participants were also recruited to participate in a related fMRI study in which group homogeneity is very important. We conducted the fMRI experiment to investigate the neuronal responses to the same tastes. The fMRI sessions took place 1 day before and 1 day after the six-day period. The results from these fMRI sessions will be reported separately (see e.g. [18]).

#### Ethics statement

The Medical Ethical Committee of the University Medical Center Groningen approved this study and informed written consent was obtained from the participants before testing.

#### Materials

The drinks were divided in two groups. The first group contained six ONS products. All six ONS products were milk based and could be subdivided in 3 products for generally underfed patients (flavors: apricot, vanilla and neutral, 160 Kcal/100 ml) and 3 products specifically aimed at underfed cancer patients (flavors: peach-ginger, cappuccino and orange-lemon, 160 Kcal/100 ml). The difference between both product groups lies both in their macro and micronutrients. However their energy content was equal. The second group of drinks consisted of eight products that were commercially available in Dutch supermarkets while conducting the experiment. The drinks can be subdivided in two groups: four water-based drinks (flavors: apple-blueberry 27 Kcal/100 ml, apple-peach 28 Kcal/100 ml, orange-tangerine 27 Kcal/100 ml and pineapple-mango 28 Kcal/100 ml) and four yogurt drinks (flavors: raspberry 33 Kcal/100 ml, coconut 32 Kcal/100 ml, lemon 33 Kcal/100 ml and orange-cinnamon 30 Kcal/100 ml). A more detailed description about the contents of the drinks can be found in Table S1.

#### Design & Procedure

The study was set up as a repeated exposure experiment in which participants were repeatedly exposed to different drinks. During the repeated exposure every participant visited the lab on 8 consecutive days. On day 1 and day 8 the participants underwent an fMRI scan session in which they tasted all their group-specific drinks three times in random order. As mentioned before, the fMRI results will not be discussed here. On day 2 to day 7 the participants were invited to visit the lab and taste the group-specific drinks. We will henceforth refer to this period as “day 1 to day 6”.

Participants were randomly assigned to two groups: a morning group and an afternoon group. The former tasted eight supermarket drinks in the morning between 8∶00 and 10∶00. The latter tasted six ONS drinks between 16∶00 and 18∶00. Both groups were instructed to fast for two hours before every experimental session (except for drinking water). During the taste and rate sessions the participant was seated in front of a table with six or eight unlabeled transparent cups, for the ONS and supermarket groups respectively. The participant was then instructed to taste every drink (from left to right, *ad libitum* up to a maximum of 100 ml) and rate the perceived pleasantness on a Likert scale ranging from 1 (not at all pleasant) to 7 (extremely pleasant). Before tasting every drink, each participant was instructed to rinse his mouth with a sip of 5% artificial saliva (Saliva Orthana, TM) diluted in water (see Table S1).

To minimize carry-over effects the drinks’ presentation order was randomized per and between participants, balanced within participants and counter balanced between participants.

### Study 2: Snacks

For the second study, we used the data from [12] (with permission). These authors investigated 1) how complexity and intensity of snacks and soups affected sensory specific satiety (SSS) and 2) the predictive value of SSS for product acceptance during repeated exposure. In the next method section, we will summarize the methods of the snacks study. For more detailed information, we refer the reader to [12].

#### Participants

Data from 53 healthy consumers (μ_age_ = 23.9, σ_age_ = 6.7, n_male_ = 25) were used in the analysis. These participants were naïve to the study and were told to assess different food prototypes.

#### Materials

Four snacks were included in this study: 1) a candy bar with chocolate and nuts (458 kcal/100 g), 2) a whole meal biscuit with chocolate (458 kcal/100 g), 3) plain (dark) chocolate (570 kcal/100 g), and 4) a tea biscuit, which is a plain wheat biscuit (437 kcal/100 g).

#### Design & Procedure

The study was set up in two parts: a SSS paradigm and a repeated exposure paradigm over 5 days. The data of the repeated exposure paradigm was used for our analysis. Within this paradigm, participants were instructed to swallow a mouthful of each product (equaling 275 kcal and 450 kcal per snack for female and male participants respectively) and to rinse with water and crackers between the samples, on day 1 to day 4. On day 5, participants were allowed to consume the products *ad libitum*. For our analysis we used the corresponding perceived pleasantness ratings. These were given on a visual analogue scale ranging from left (not at all pleasant) to right (extremely pleasant).

The data of the Snacks Study differs from the Drinks Study with respect to product types (snacks versus drinks), context (different study) and rating scale (10 cm line scale versus 7-point Likert scale).

### Statistical Analysis

All analyses were performed in R (www.r-project.org, version 2.14.2, 2012-02-29). In the Drinks Study dataset, 76 (4.01%) observations were randomly missing. These missing observations were imputed using the *k*-nearest neighbor method (kNN) [19] provided via the kNNImpute function from the package Imputation (version 1.3). The optimal value of *k* was found via cross validation. In the snacks study, no data were missing.

To investigate the temporal liking patterns, we fed all participant-drink combinations to the analysis. The data set of the Drinks Study consisted of 316 participant-drink combinations (22 participants tested 6 drinks and 23 participants tested 8 drinks). The data set of the Snacks Study consisted of 212 participant-snack combinations (53 participants tasted 4 snacks).

We used principal component analysis (PCA) for giving graphical insight in the different participant-drink behaviors over time. The consecutive days in the repeated exposure paradigms functioned as the variables on which the PCA transformation was performed. For both studies we will show the biplot of the first 2 principal components and report the variance explained.

To find typical temporal liking dynamics in the dataset we used the cluster algorithm *k*-means [20]. *K*-means finds the maximum distance between the centers of *k* groups and minimizes the within-distance inside clusters at the same time [21]. To minimize the risk of local minima [22], clustering was repeated 100 times and the solution with the smallest within group sums of squares (WSS) was used. The optimal number of clusters can be determined by algorithmic, graphical and formulaic methods [23]. We used a set of four methods described in [23] to estimate the best value of *k*: two algorithmic methods that calculate the Bayesian Information Criterion (BIC) [24] implemented for k-means based either on maximum likelihood estimation [25] (method 1) or on the maximum a posteriori (MAP) estimator provided in package mclust (version 3.4.11) [26], [27] (method 2), a graphical method that finds the value of *k* at the bend within the WSS plot [28] (method 3), and the formulaic method the Gap statistic [29] (method 4). For an overview of the outcomes of these methods on both data sets, see Table S2.

In order to investigate cluster intercepts (i.e. level of liking) and cluster slopes (i.e. temporal dynamics of liking) we modeled the temporal liking behaviors with maximum-likelihood-based linear mixed models (LMM). For all models, liking scores were entered as dependent variable, while cluster identity and the interaction between cluster identity and time in days (i.e. the cluster slope over time) constituted the fixed variables in the model. The subjects were included as random variable. LMMs are provided via the lmer-function from package lme4 (version 0.999375-42) [30]. We will report p-values as well as upper and lower 95% highest posterior density (HPD95) intervals obtained by Markov Chain Monte Carlo (MCMC) sampling (10,000 samples, using the package languageR, version 1.4) [31]. The HPD95 intervals can be interpreted as traditional 95% confidence intervals and mark the expected range of the underlying parameter. For a discussion on similar analysis techniques, see [32].

For all reported LMMs, we will also report the quality of fit statistic, denoted as explained deviance (D). The amount of explained deviance by each model term is the sum of squares of a fixed-effect divided by the sum of squares total, which is calculated here as the variance of the independent variable multiplied by its length. The calculation is provided in the package LMERConvenienceFunctions (version 1.6.8.2) [33]. We used the post hoc procedure from the same package to estimate the intercepts and slope per individual cluster (using 10,000 MCMC samples). Note that the rating scales are always positive. Therefore, cluster intercepts will always deviate from zero, resulting in obvious p-values for cluster intercepts.

## Results

### Study 1: Drinks Data

**Figure 1:**
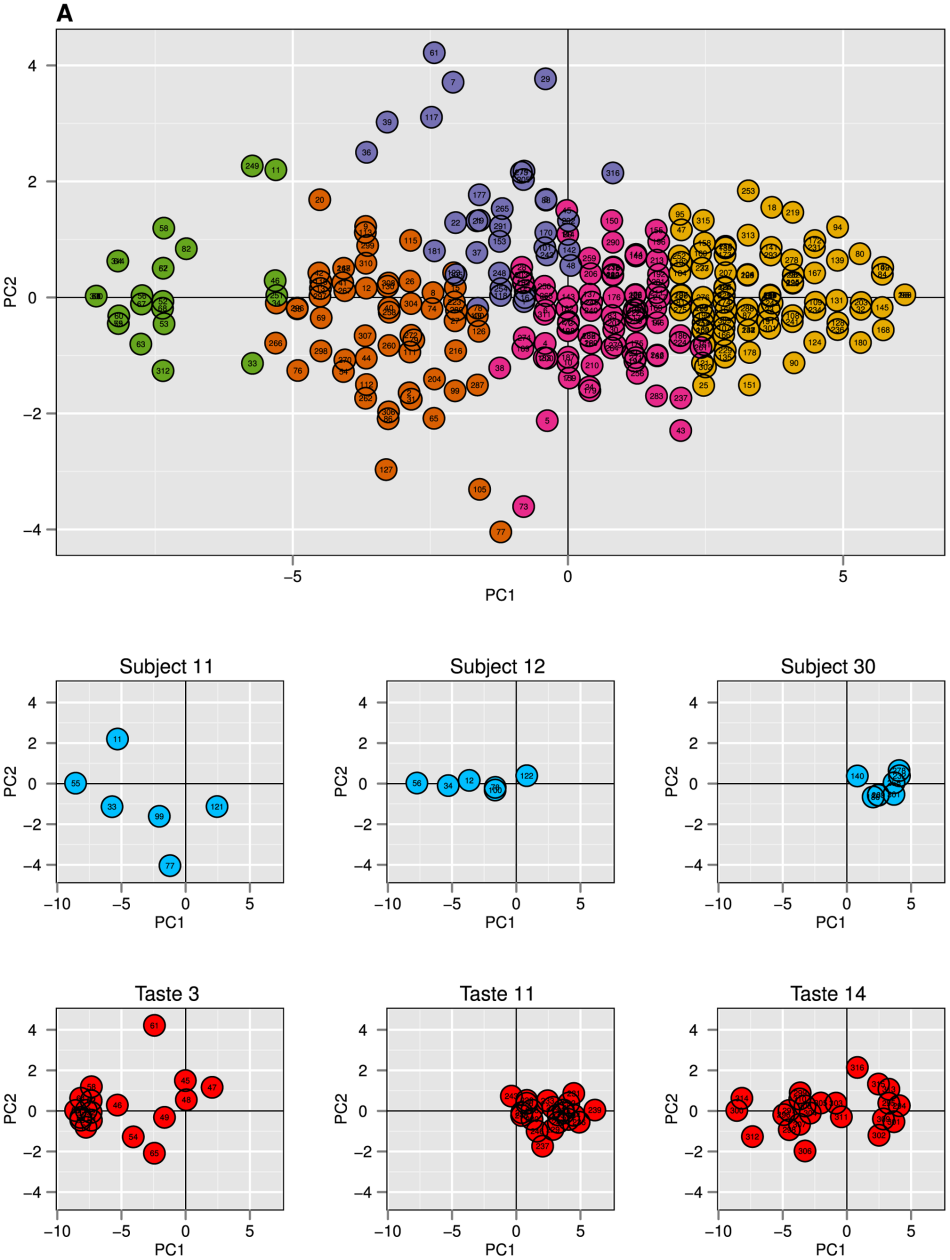
PCA analysis on repeated exposure data from the Drinks Study. **A**) Biplot representing the first and second principal component of 316 participant-product combinations that contained liking ratings over 6 days. These first 2 principal components account for 83% of the variance. Every data point represents the rating behaviour of one participant-product combination over 6 days. The colors represent 5 clusters that were generated with k-means clustering on the raw data. Manual inspection showed that PC1 captured the level of liking during the six days of repeated exposure. Ranging from extremely dislike (left) to extremely like (right). PC2 captured the temporal dynamics of liking. Ranging from a decline in liking over time (down) to an increase in liking over time (up). **B**) Three examples of specific subjects. The data points represent their assigned products. **C**) Three examples of specific drinks. The data points represent all participants that were repeatedly exposed to these products.

Figure 1A provides an overview of the temporal liking behaviors in a PCA biplot. Every observation in the biplot represents the rating pattern of a single participant-product combination over six days projected on the first 2 principal components. Manual inspection showed that the first principal component (PC1, 76% variance explained) captures the level of liking during the six days of repeated exposure. Ranging from extremely dislike (left) to extremely like (right). The second principal component (PC2, 7% variance explained) captured the temporal dynamics of liking. Ranging from a decline in liking over time (down) to an increase in liking over time (up).

Figure 1B shows three examples of subject-specific behaviors extracted from the PCA result in Figure 1A. The observations represent all products that were tasted by a single participant. The three examples show different types of subject-behavior. Subject 11 varied highly in his temporal rating dynamics on the products (PC2) and disliked the majority of the products (negative scores on PC1). Subject 12 rated the products very consistently over time (low variance on PC2) and clearly ordered them from dislike to moderately like (incremental changes on PC1). Subject 30 basically equally liked all products and rated them consistently over time (low variance on both PC1 and PC2).

Figure 1C shows three examples of product-specific behavior (group results). Product 3 (ONS neutral) is a mainly extremely disliked product (negative tight cluster on PC1), where most participants showed fairly stable disliking over time in this cluster (low variance on PC2). Product 11 (Supermarket Raspberry yogurt drink) was a liked product (mainly positive scores on PC1) with little temporal dynamics (scores on PC2 around 0). Product 14 (Supermarket Orange-Cinnamon) turned out to be a bimodal product with a liker group (positive cluster on PC1) and a slightly larger disliker group (negative cluster on PC1). For a complete overview of subject-specific and product-specific behaviors within this data set, see Figures S1 and S2.

**Figure 2:**
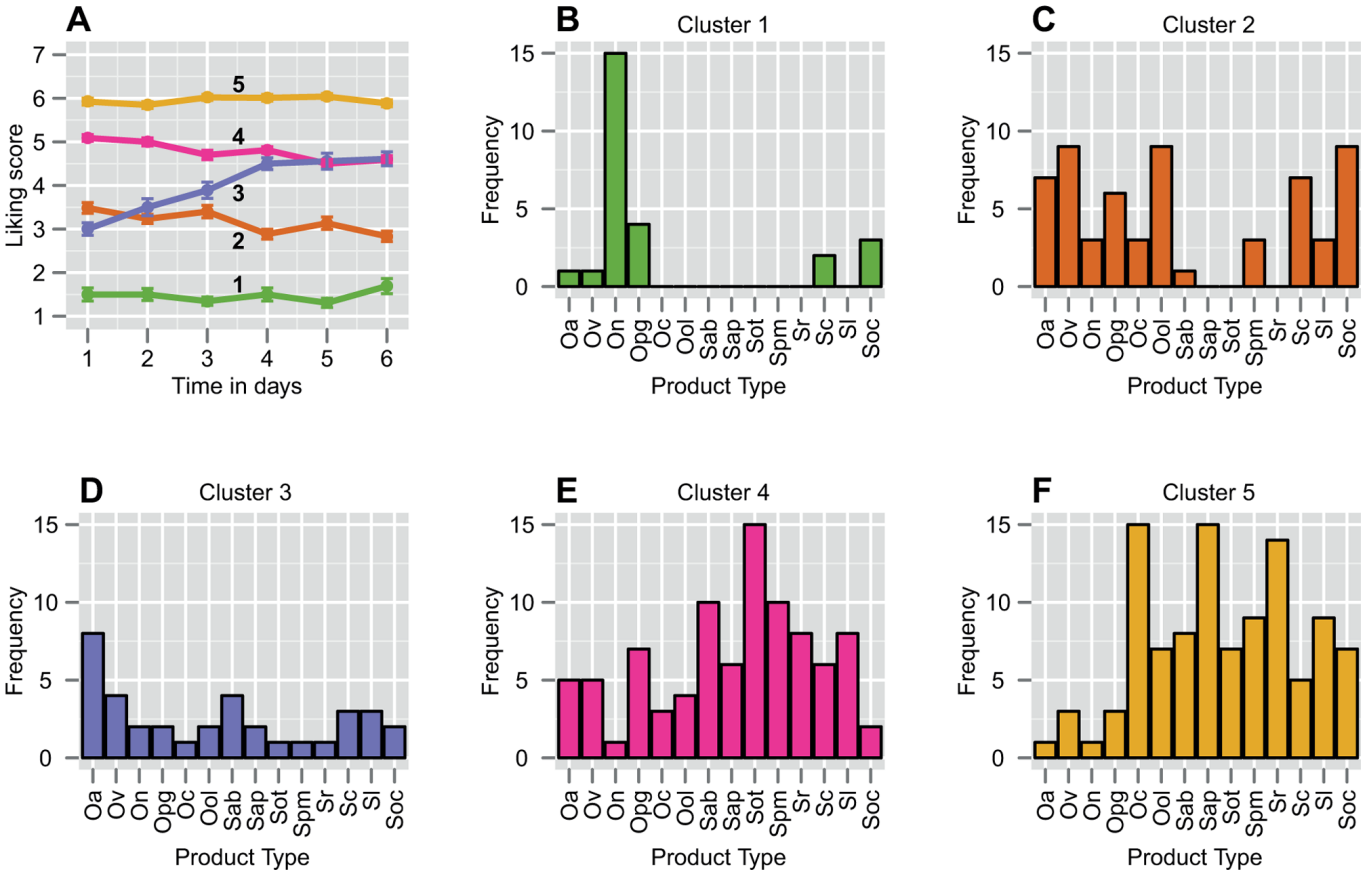
Characteristic liking behaviors and product-cluster assignment in the Drinks Study. **A)** The five characteristic liking behaviors over time generated by *k*-means clustering on liking scores during six days of repeated exposure in the Drinks Study. Clustering was done on 316 participant-product combinations. The error bars indicate the SEM. The numbers in bold refer to the cluster numbers. **B–F)** Frequency plots showing how often every drink was included in every cluster. The color coding of the frequency plots refer to the color coding of the clusters in **A**, ranging from the cluster with the lowest scores (**B**) to the cluster with the highest scores (**F**). The ONS drinks are apricot (Oa), vanilla (Ov), neutral (On), peach-ginger (Opg), cappuccino (Oc) and orange-lemon (Ool). The supermarket drinks are apple-blueberry (Sab), apple-peach (Sap), orange-tangerine (Sot), pineapple-mango (Spm), raspberry (Sr), coconut (Sc), lemon (Sl) and orange-cinnamon (Soc).

We used *k*-means to find homogenous groups of liking behaviors over time. The methods to estimate the value of *k* (number of clusters) showed little variation in their optimal outcomes; 4< = *k* < = 6 (see Table S2). Visual inspection showed that the choice of 4 clusters did not provide any information about the temporal dynamics, while 6 clusters seemed to overfit the data by introducing a new fairly noisy cluster. Therefore, *k* was set to 5 for further analysis (see Figure S3, for the results of 4,5 and 6 clusters). The result of the five clusters can be found in PCA biplot of Figure 1A, where all 5 clusters are indicated with a different color. Figure 2A shows the characteristic temporal behaviors captured in these five clusters. The 5 frequency plots in Figure 2B–F indicate how often every taste contributed to the specific cluster.

A LMM (Table 1, D = 63.9%) was constructed to estimate the intercepts and slopes of the clusters. The result revealed two stable clusters (β_intercept cluster 1_ = 1.49; β_intercept cluster 5_ = 5.91) on the extremes of the scale that showed no significant temporal changes. Furthermore, a cluster within the liking range (β_intercept cluster 4_ = 5.18) and a cluster in the disliking range (β_intercept cluster 2_ = 3.59) showed a significant decline in liking over time (β_slope cluster 2_ = −0.12, p<0.01; β_slope cluster 4_ = −0.11, p<0.001). Finally, one cluster in the disliking range (β_intercept cluster 3_ = 2.84) showed a significant increase in liking over time (β_slope cluster 2_ = 0.34, p<0.001).

**Table 1:** Cluster statistics from the Drinks Study.

Cluster	Intercept β	Intercept HPD95	Intercept p_MCMC_	Slope β	Slope HPD95	Slope p_MCMC_	N^1^	N unique^2^
1	1.49	1.20, 1.79	<0.001	0.006	−0.07, 0.07	0.86	26/316	21/55
2	3.59	3.38, 3.80	<0.001	−0.12	−0.16, −0.06	<0.01	60/316	34/55
3	2.84	2.58, 3.10	<0.001	0.34	0.27, 0.41	<0.001	36/316	27/55
4	5.18	5.01, 5.35	<0.001	−0.11	−0.15, −0.07	<0.001	90/316	37/55
5	5.91	5.75, 6.07	<0.001	−0.01	−0.03, 0.05	0.60	104/316	38/55

1 Number of participant-drink combinations per cluster.

2 Number of unique participants per cluster.

The cluster statistics for five clusters based on mixed effect linear modeling and their respective cluster size details. The results are based on the Drinks Study.

Table 1 also indicates the number of participant-product combinations per cluster and the total unique participants per cluster. As expected, the majority of the participant-product combinations are in the higher clusters (4 and 5), because most products are successful off-the-shelf products, created to be as pleasant as possible. The distribution of participants across clusters was such that at least 38% (21 out of 55) of all participants are represented in a single cluster.

### Study 2: Snacks Data

**Figure 3:**
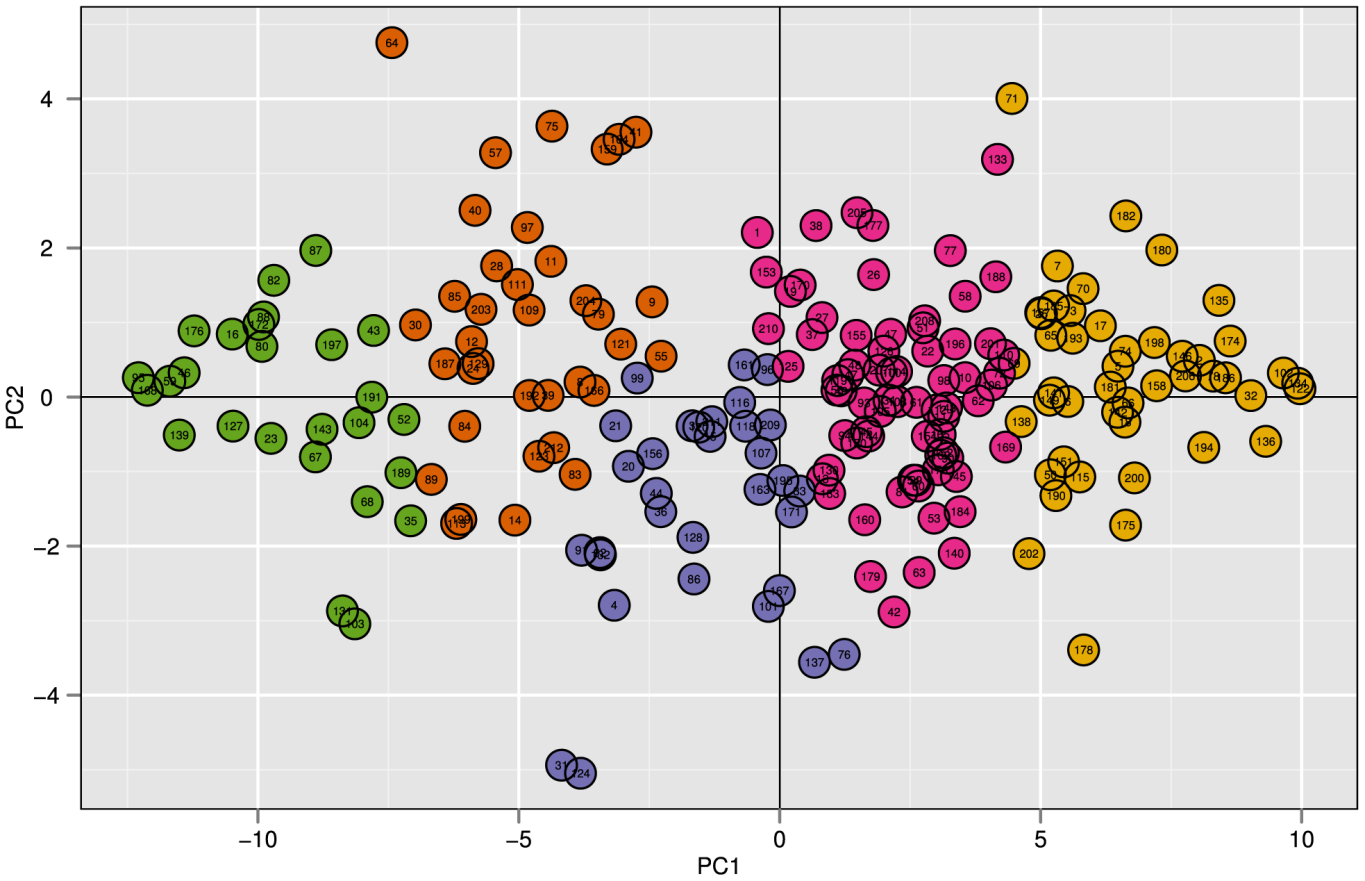
PCA analysis on repeated exposure data from the Snacks Study. Biplot representing the first and second principal component of 212 participant-product combinations that contained liking ratings over 5 days. These first 2 principal components account for 92% of the variance. Every data point represents the rating behaviour of one participant-product combination over 6 days. The colors represent 5 clusters that were generated with *k*-means clustering on the raw data. Manual inspection showed that PC1 captured the level of liking during the six days of repeated exposure. Ranging from extremely dislike (left) to extremely like (right). PC2 captured the temporal dynamics of liking. Ranging from a decline in liking over time (down) to an increase in liking over time (up).

To investigate the repeatability of our findings, we repeated the analyses on an existing repeated exposure study on snacks [12]. A PCA biplot of the first two principal components is given in Figure 3. Analogously to Figure 1A in the Drinks Study, manual inspection again showed that the first principal component (85% variance explained) captures the level of liking (extremely dislike to extremely like), while the second principal component (7% variance explained) captures temporal dynamics of liking (i.e. positive, negative and no slope).

The data was again clustered with *k*-means. As in the previous experiment, the methods to estimate *k*, indicated that a range of k (2< = *k* < = 6) provides an optimal description of the data. For similar reasons as in the Drinks Study, we continued the analysis with five clusters. The cases of 4, 5 and 6 clusters are shown in Figure S4.

**Figure 4:**
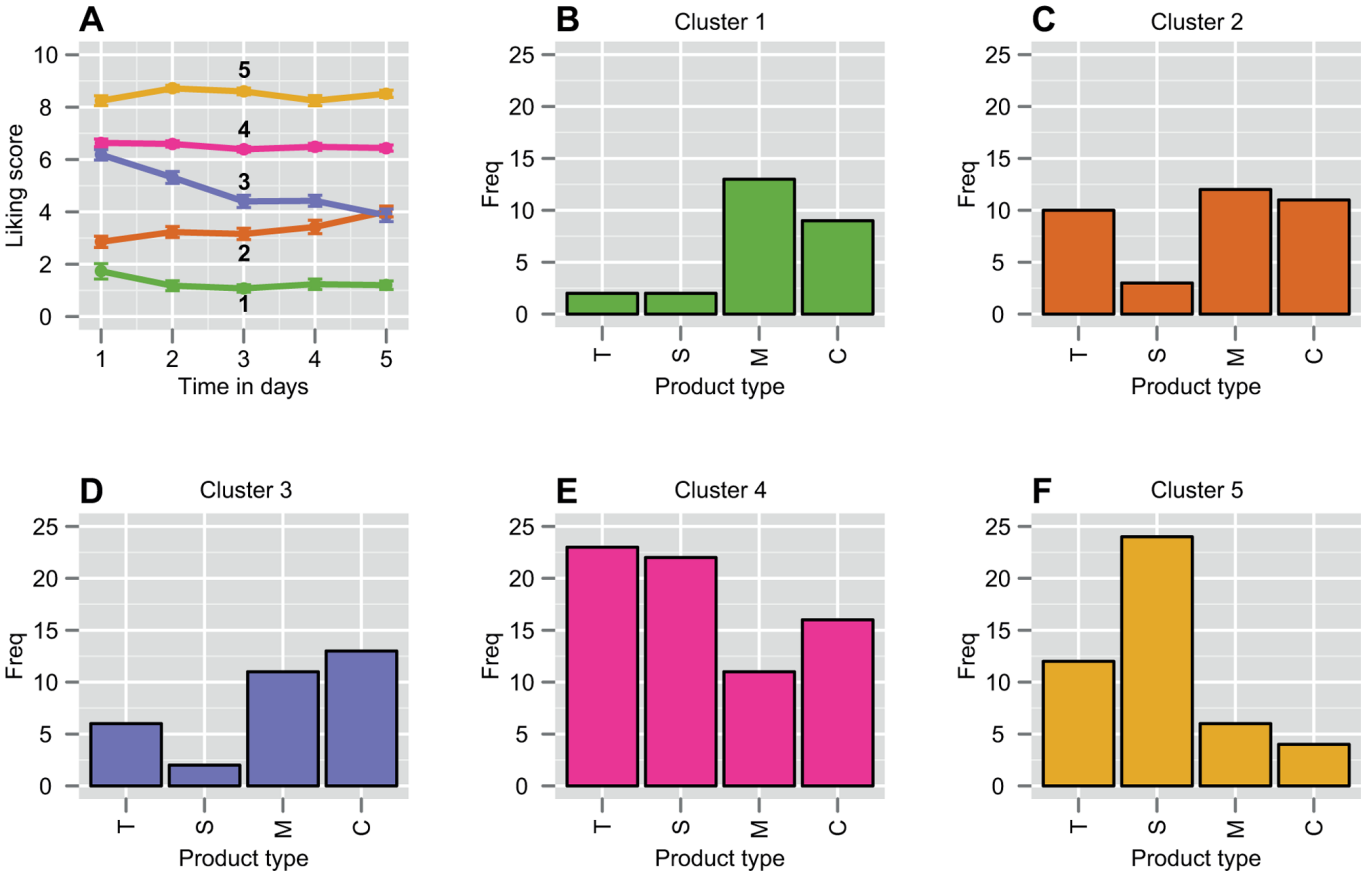
Characteristic liking behaviors and product-cluster assignment in the Snacks Study. **A)** The five characteristic liking behaviors over time generated by *k*-means clustering on liking scores during six days of repeated exposure in the Snacks Study. Clustering was done on 212 participant-product combinations. The error bars indicate the SEM. The numbers in bold refer to the cluster numbers. **B–F)** Frequency plots showing how often every drink was included in every cluster. The color coding of the frequency plots refer to the color coding of the clusters in **A**, ranging from the cluster with the lowest scores (**B**) to the cluster with the highest scores (**F**). The products were; a tea biscuit (T), a candy bar with chocolate and nuts (S), a whole meal biscuit with chocolate (M) and plain chocolate (C).

Figure 4A shows the characteristic temporal behaviors captured in these five clusters. The five frequency plots in Figure 4B–F indicate how often every taste contributed to the specific cluster.

For the Snacks Study, we constructed another LMM to estimate the intercepts and slopes of all five clusters. The outcome is summarized in Table 2 (D = 70.51%). The model indicated that besides the clusters on the extremes of the scale (β_intercept cluster 1_ = 1.57; β_intercept cluster 5_ = 8.42) another cluster within the liking range (β_intercept cluster 4_ = 6.68) showed no significant temporal changes. One cluster within the liking range (β_intercept cluster 3_ = 6.56) showed a significant decline in liking over time (β_slope cluster 3_ = −0.58, p<0.001), ending in the disliking range after 5 days. Finally, one cluster in the disliking range (β_intercept cluster 2_ = 2.57) showed a significant increase in liking over time (β_slope cluster 2_ = 0.25, p<0.001). The distribution of participants across clusters indicated that at least 42% (22 out of 53) of all participants are represented in a single cluster.

**Table 2:** Cluster statistics from the Snacks Study.

Cluster	Intercept β	Intercept HPD95	Intercept p_MCMC_	Slope β	Slope HPD95	Slope p_MCMC_	N^1^	N unique^2^
1	1.57	1.01, 2.02	<0.001	−0.1	−0.24, 0.04	0.15	26/212	45/53
2	2.57	2.20, 2.97	<0.001	0.25	0.13, 0.37	<0.001	36/212	29/53
3	6.56	6.14, 6.96	<0.001	−0.58	−0.70, −0.46	<0.001	32/212	28/53
4	6.68	6.34, 6.96	<0.001	−0.05	−0.13, 0.03	0.23	72/212	22/53
5	8.42	8.07, 8.77	<0.001	0.006	−0.09, 0.11	0.91	46/212	26/53

1 Number of participant-drink combinations per cluster.

2 Number of unique participants per cluster.

The cluster statistics for five clusters based on mixed effect linear modeling and their respective cluster size details. The results are based on the Snacks Study.

## Discussion

In the current study, we investigated how to deal more effectively with interindividual differences in liking during repeated exposure to food and how these differences can be characterized. Previous studies showed that consumer liking could potentially change over time; liked products do not necessarily stay liked and disliked products do not necessarily stay disliked. However, to the best of our knowledge product liking is mainly analyzed on a product level by averaging responses of all participants per product. Consequently, interindividual differences will be ignored, which could lead to a misinterpretation of the results.

A good example of possible dynamics in liking is shown by Weijzen et al. [12]. The authors nicely show that temporal dynamics in consumer liking towards a whole meal biscuit with chocolate can be revealed by means of a repeated exposure experiment; this product is neutrally liked and becomes more liked over time (see Fig. 3 in [12]). However, can we conclude that a “typical consumer response” to this product would result in an increase in liking during repeated exposure, *ranging from neutral to moderately like?* Our proposed refinement to analyzing repeated exposure to foods indicated that this is not the case. We showed that an increase in liking was present in a group of participants that resided in cluster 2: *ranging from dislike to moderately dislike,* leading to the conclusion that this product becomes less disliked instead of more liked for this specific group of participants. Moreover, our cluster analysis showed that this product elicited very different liking behaviors in multiple subgroups of the participants.

### Characteristic Liking Behaviors

Previous studies indicated that an increase in liking towards food was mostly found when the food was initially neutrally liked or moderately disliked [2]–[5], [34]. For initially liked products however, liking either remained stable or decreased over time, where the latter could be attributed to boredom or loss of interest [6], [7], [9], [12], [13], [15].

In the current study, we proposed to identify and analyze temporal liking behaviors that occur both within and between participants over all products under investigation: the participant-product combinations. *K*-means clustering unveiled five characteristic liking behaviors in two separate studies containing drinks and snacks. We showed that two different data sets contained five clusters with some small differences in liking behavior. In both data sets a cluster was identified in which liking increased over time, when the product was initially disliked or neutrally liked. Clustering also showed characteristic boredom/loss of interest behavior. This behavior not only occurred when the taste was initially liked (Drinks Study), but also when the taste was initially disliked (Snacks Study). Furthermore, we found that liking for products, which are initially extremely liked or extremely disliked, tended to stay stable over time. This means that initially liked products do not necessarily have to suffer from boredom during repeated exposure and that previously found boredom effects could be due to an end-scale effect; liking can only decrease.

In previous research, Kremer et al. (2013) and Hoek et al. (2013) showed that by categorizing the possible directions of the liking slope during repeated exposure, consumers could be segregated in groups that show a positive change, a negative change or no change in liking over time. Our results show that we were able to extend this work by segregating characteristic consumer behaviors not only on differences in slope (i.e. temporal dynamic), but also on differences in intercepts (i.e. level of liking) (see Tables 1 & 2).

### Product Modality

Köster [16] suggested that liking on products is possibly uni-, bi- or multimodal and that often little attention is paid to these underlying liker groups. Based on the product frequencies per cluster (Figures 2B–F & 4B–F) and the product-specific behaviors (Figure, 1C), we found that products in both the Drinks and Snacks study are indeed uni-, bi-, or multimodal. Two unimodal products in the Drinks Study were the ONS neutral and the ONS cappuccino. In both cases, 68% of the participants who tasted these drinks concentrated in one cluster, mainly disliking the former and liking the latter. A fairly unimodal product in the Snacks Study was the candy bar with chocolate and nuts. Participants showed moderate to extreme liking to this product and mainly occupied clusters 4 and 5 (87% in total). In their study, Weijzen et al [12] showed that perceived complexity positively affects sustained acceptability. The candy bar with nuts stood out from the other snacks because it was perceived as the most complex. A strong bimodal product was the Orange-Cinnamon drink where 41% of participants occupied the clusters with positive liking (4 & 5) while 50% ended up in the clusters showing negative liking (1 & 2). The whole meal biscuit with chocolate could be regarded as a multimodal product, where 20–25% of the participants occupied each of the first four clusters, while 11% extremely liked the product. These results show that we could indeed find product modality in both data sets. Therefore, we confirmed the modality hypothesis by Köster in a repeated exposure context.

### Consumer Consistency

Almost all studies on repeated exposure to food products indicate that (groups of) consumers change their evaluation during the course of the exposure. Lévy and Köster [35] and Köster et al. [36] discussed the issue of consumer consistency and indicated that changes in consumer preferences even occur within a tasting session. Therefore, Lévy and Köster [35] concluded that one hedonic measurement is often a poor indicator for future liking but “*that a 3 day exposure … suffices to find a remarkable change in final choice*”. Although we agree with Lévy and Köster [35] that one measurement is insufficient to effectively determine future liking, we would argue that temporal dynamics of liking within certain clusters still change remarkably between day 3 and 4 (see Figures 2 & 4). Therefore, we propose to extend the repeated exposure period to at least 4 days of testing in order to draw better conclusions about future liking.

Our results suggest that there is also consumer inconsistency in rating strategy. The subject-specific examples in Figure 1B clearly show that consumers apply different strategies for rating the range of products; some subjects show high variety in their liking between products and over time, while other subjects clearly order the products or rate everything equally. This result suggests that consumers can show rating inconsistency in multiple ways within an experiment: not only in single product ratings but also in rating strategy.

### Using Clustering to Identify Temporal Liking Patterns

A different method of analyzing the liking patterns is selecting consumer-product combinations based on initial liking. However, when temporal patterns are investigated after an initial selection based on measurement scores, the result will generally show a regression to the mean effect [37]. Regression to the mean can also not be excluded when products are selected based on initial liking [38]. An advantage of clustering is the insensitivity to this selection bias. Therefore, we used this method to identify characteristic temporal patterns in liking in the current study.

A disadvantage of clustering is the uncertainty about the true number of clusters within a data set. The determination of the best number of clusters (*k*) is a standing issue. We showed that different methods to determine *k*, did not agree and indicated a range of 2 to 6 clusters in the extreme case. Based on previous research we expect consumers to change their liking over time, whether it be a boredom effect or a repeated exposure effect. Because, the use of 2,3 or 4 clusters did not provide any information about these expected temporal dynamics of liking and while 6 clusters seemed to overfit the data, we chose to use 5 clusters in both studies (see Figures S3 and S4 for results on this topic).

### Stimulus Range

The range of stimuli during a repeated exposure experiment inherently determines the clustering result. Our results suggest that consumers tend to behave accordingly to one out of five prototypical behaviors when repeatedly consuming a food product. When the stimulus range solely consists of mostly liked products or mostly disliked products, one or several clusters may not be present. In this case a different number of clusters would be more appropriate.

Some research indicates that the stimulus range could form a bias. A hedonic judgment on a specific taste stimulus is influenced by stimuli in the same context; a problem known as the stimulus range effect [39]–[41]. We performed our analyses on two independent data sets. Although the products in both studies were all fabricated to be pleasant, clustering these data sets showed a similar division in characteristic temporal liking behaviors between the Drinks and Snacks study, covering the entire rating scale. Therefore, we have no reason to believe that the stimulus-range effect influenced the identification of the characteristic temporal liking behaviors.

For future product testing we would argue to use a range of products in repeated exposure paradigms for two reasons. Firstly, including multiple products allows defining the characteristic temporal liking behaviors that are present within the specific dataset. These characteristic behaviors form a reference frame for identifying product modality. Including a balanced set of known liked and disliked products could optimize this reference frame. Secondly, a larger product range increases variety, which reduces product boredom caused by the experiment itself. When participants are forced to consume one and the same product for multiple occasions, product disliking will be more likely to be induced by the monotony of the experimental paradigm itself. Data partially confirming this hypothesis comes from Zandstra et al. [14]. These authors showed that participants reported lower product boredom when variety was offered between sessions.

### Liking and Intake

This study was set up to characterize different consumer behaviors in liking. For data collection in the drinks study, we intended to induce these liking behaviors within an ecological valid repeated exposure context. Therefore we not only included a wide range of commercially available complex taste stimuli that ranged from generally disliked to generally liked, but also allowed for *ad libitum* intake. Arguably, intake can affect liking behavior. Weijzen et al., [12] showed that liking and intake were highly related in the snacks study (r = .65). However, we argue that introducing intake as covariate would mitigate effects of liking in our analyses.

### Future Perspectives

The finding that liking changes during repeated exposure is not limited to gustatory or olfactory stimuli alone. The effects are also reported for e.g. visual and auditory stimuli [42]–[44]. It would be interesting to see whether similar characteristic temporal dynamics also underlie these senses and whether a general neuronal system can be found that underlies specific liking behavior(s).

Furthermore, it would be interesting to link cluster membership to personality, age, gender and social-economical status; what are the characteristics of a consumer subgroup that shows a specific liking behavior on a product of interest? Knowing these characteristics could help aiming this product specifically to a consumer group.

### Conclusion

We showed that there is great variability in consumer liking during repeated exposure to different food products. By using multiple products and clustering consumer-product combinations we were able to unveil five clusters of liking behaviors during repeated exposure. This work extended previous work by describing differences in consumer liking more effectively in terms of both the level of liking and temporal dynamics in liking. Furthermore, cluster-participation provided insight in uni-, bi- and multimodal product liking.

## Supporting Information

Figure S1 **PCA biplot of the temporal subject dynamics in liking.** The figure shows the temporal liking data of every single subject in a biplot generated by a PCA on the complete data set of the Drinks Study. Every observation represents the loading on both PC1 and PC2 for a single product. The PCA captured the temporal liking intercept on PC1 (dislike = negative, like = positive) and the temporal liking slope on PC2 (increase in liking = positive, decrease in liking = negative). Subject 1 to 22 tasted the ONS drinks, while subject 23 to 45 tasted the supermarket drinks. The differences between subjects show both differences in liking and rating strategy.
(PDF)

Figure S2 **PCA biplot of the temporal product dynamics in liking.** Analogously to the PCA biplot as in Figure S1, the temporal liking data are given in a biplot generated by a PCA on the complete data set of the Drinks Study. However, here the observations are given per product in every plot. These plots give insight in product modality. A unimodal product shows a large single cluster of observations (e.g. products 3 and 8). A bimodal product shows two clusters of observations (e.g. products 12 and 14). A multimodal product shows a large spread of observations (e.g. product 4).
(PDF)

Figure S3 **Liking during repeated exposure in the Drinks Study for 4,5 and 6 clusters.** This figure shows the temporal dynamics of liking for 4,5 and 6 clusters in the Drinks Study. As can be seen the choice of 4 clusters will not show any information about the dynamics in time, whereas with five clusters these dynamics are visible. The choice of 6 clusters seems to overfit the data: a new fairly noisy cluster is introduced when the number of clusters increases from 5 to 6.
(PDF)

Figure S4 **Liking during repeated exposure in the Snacks Study for 4,5 and 6 clusters.** This figure shows the temporal dynamics of liking for 4,5 and 6 clusters in the Snacks Study. As can be seen the choice of 4 clusters will not show any information about the dynamics in time, whereas with five clusters these dynamics are visible. The choice of 6 clusters seems to overfit the data: the light green cluster with an intercept of ±6.5, (5 clusters), is split into two clusters (6 clusters) with intercepts of ±6 and ±7 respectively, showing very little information gain.
(PDF)

Table S1 **Stimuli information from the Drinks Study.** This table contains a more detailed description of all the products that were used in the Drinks Study. The products are subdivided in their associated product family.
(DOCX)

Table S2 **Determining the number of k-means clusters for both datasets.** We used *k*-means to find homogenous groups of liking behaviors over time. The value of *k* (number of clusters) was chosen based on four different methods depicted in these tables. For BIC CL, BIC MAP and Gap Statistic the best values are in bold and indicated with a star. For WSS, the knee in the WSS plot determines the optimal number of clusters, which is an approximation based on visual inspection and indicated by X’s. As can be seen, there is little variation in the outcomes in both datasets; 4< = *k* < = 6. Visual inspection showed that the choice of 4 clusters did not provide any information about the temporal dynamics, while 6 clusters seemed to overfit the data (see Figures S3 and S4).
(DOCX)
