# Supplementary figures and images for: Dealing with Consumer Differences in Liking during Repeated Exposure to Food; Typical Dynamics in Rating Behavior
Source: PLoS One. 2014 Mar 25;9(3):e93350. doi: 10.1371/journal.pone.0093350 (PMC3965558; doi:10.1371/journal.pone.0093350)

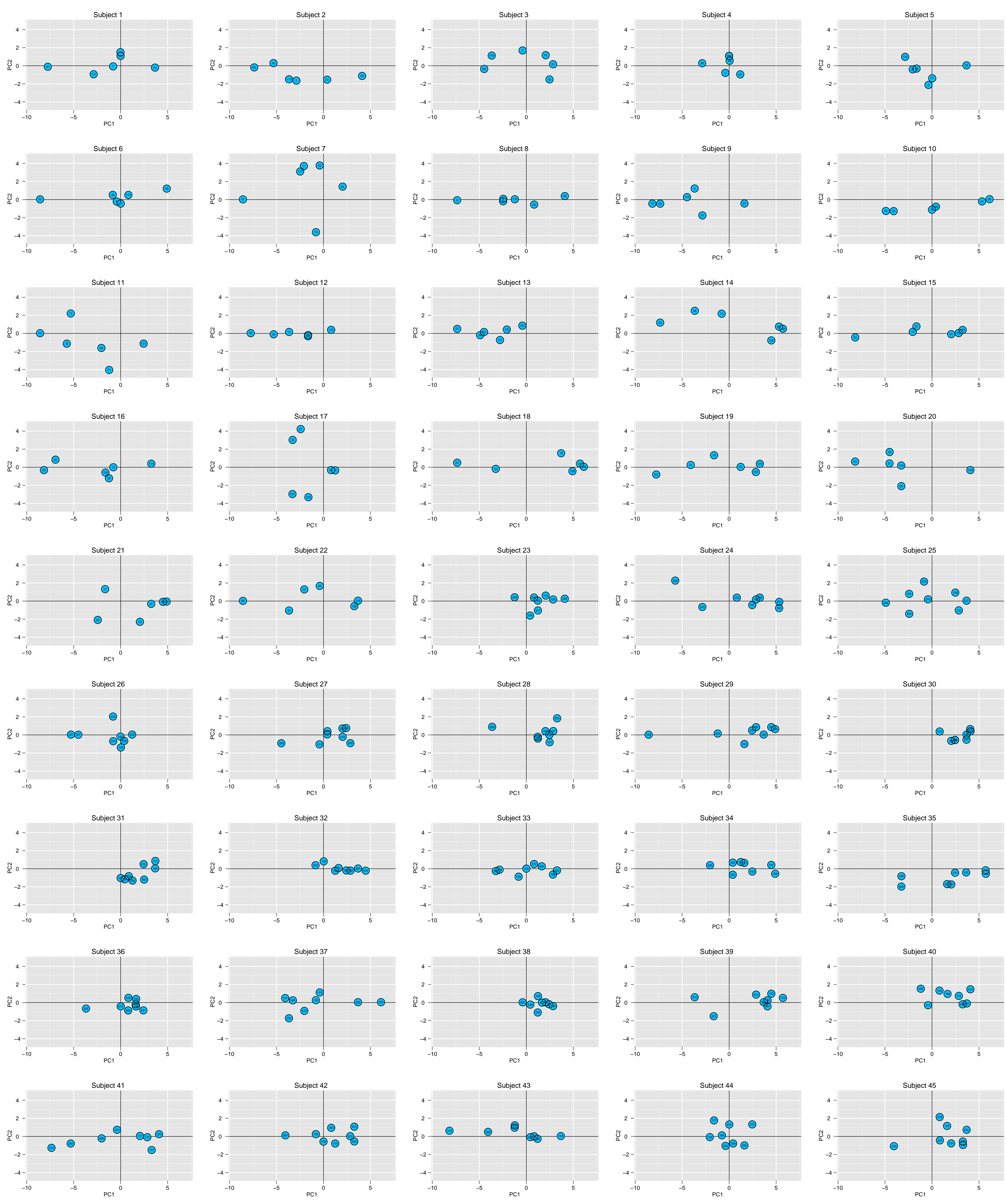

Supplement: Figure S1 — PCA biplot of the temporal subject dynamics in liking. The figure shows the temporal liking data of every single subject in a biplot generated by a PCA on the complete data set of the Drinks Study. Every observation represents the loading on both PC1 and PC2 for a single product. The PCA captured the temporal liking intercept on PC1 (dislike = negative, like = positive) and the temporal liking slope on PC2 (increase in liking = positive, decrease in liking = negative). Subject 1 to 22 tasted the ONS drinks, while subject 23 to 45 tasted the supermarket drinks. The differences between subjects show both differences in liking and rating strategy. (PDF) [file pone.0093350.s001.pdf]

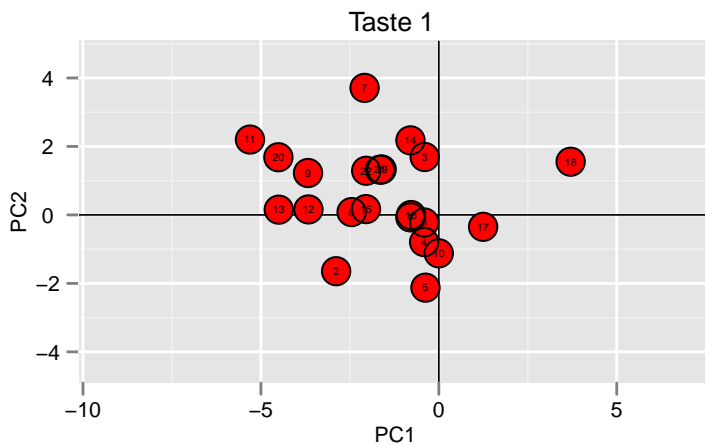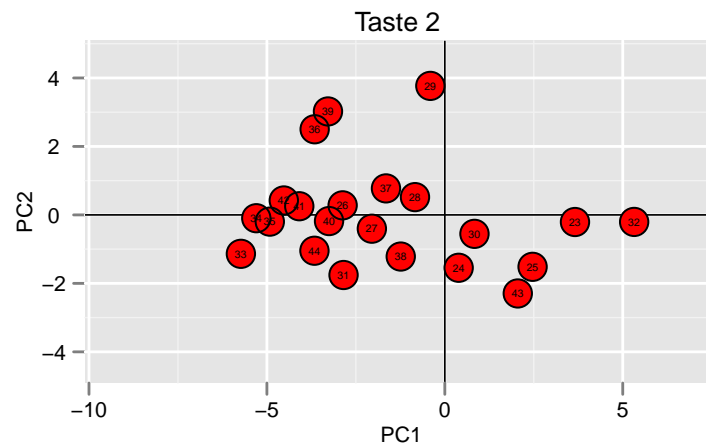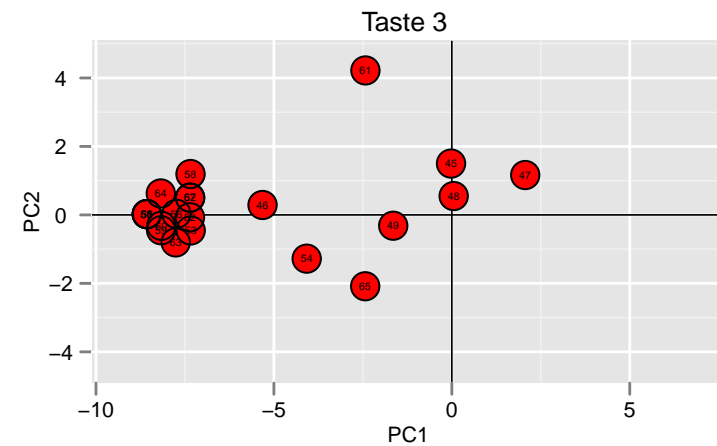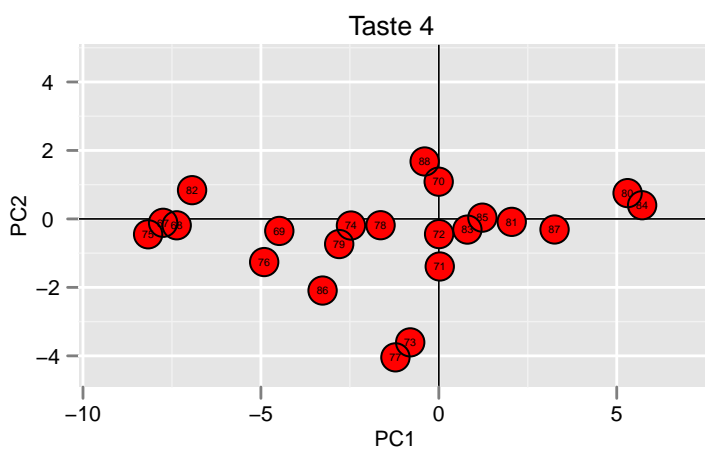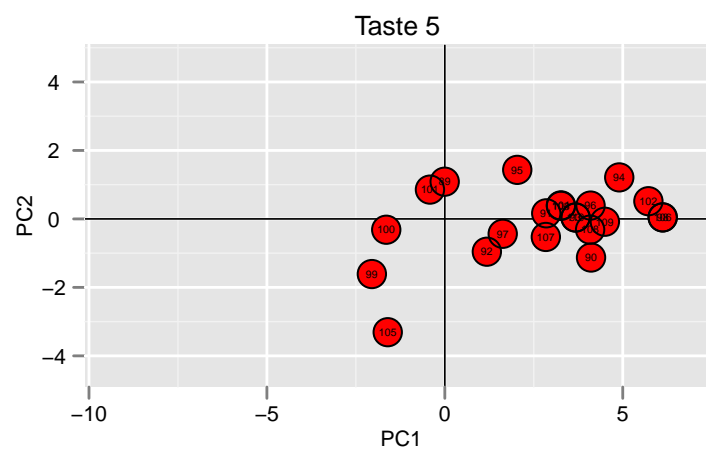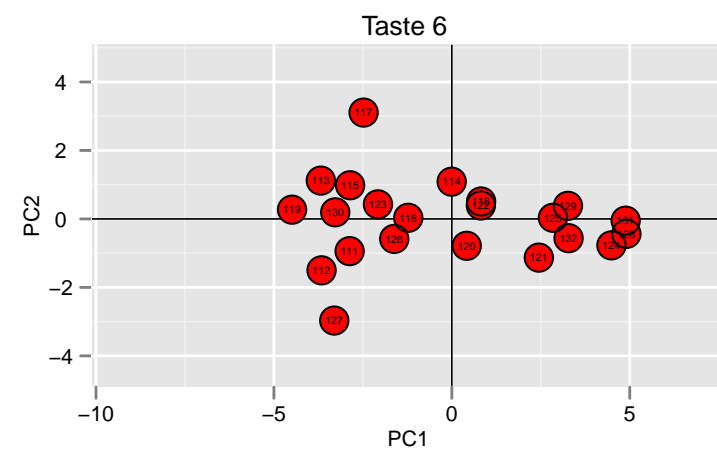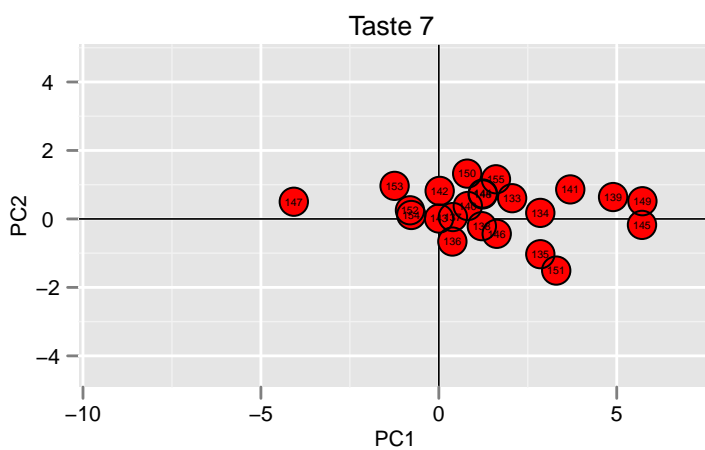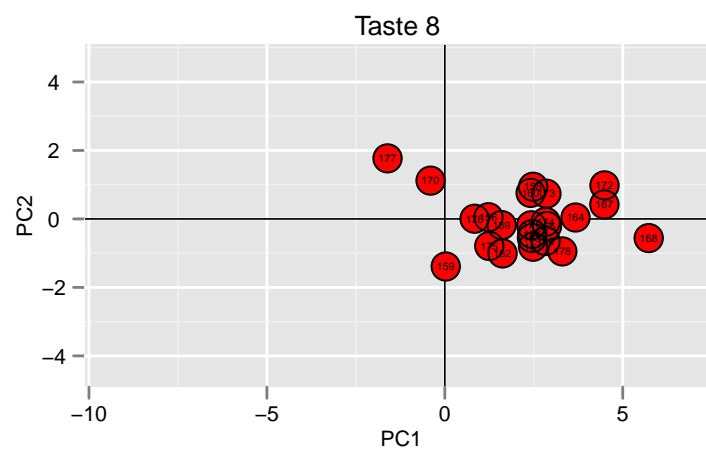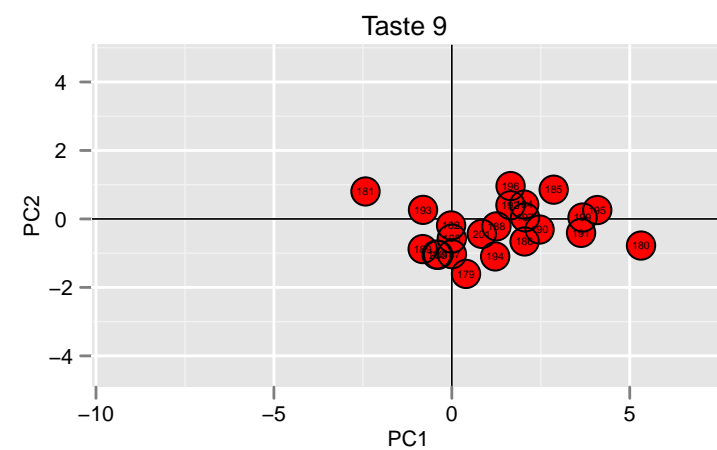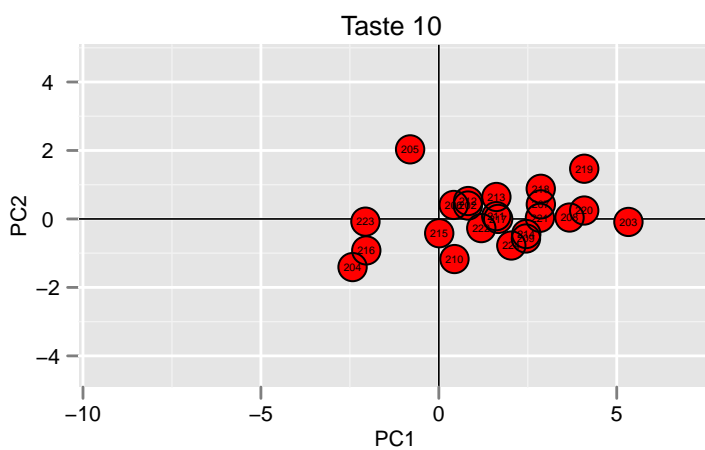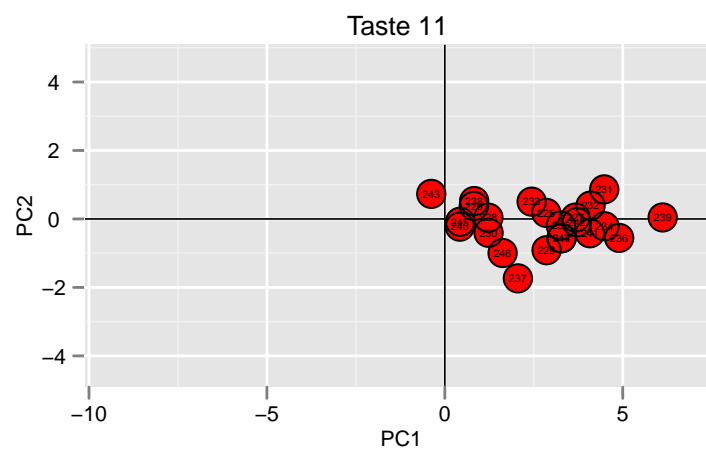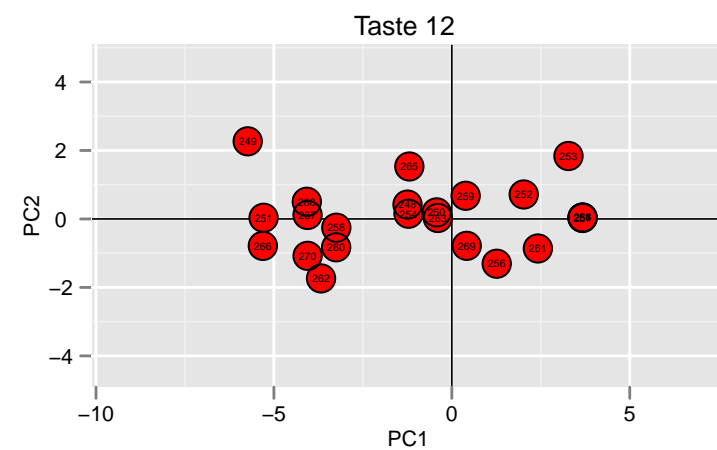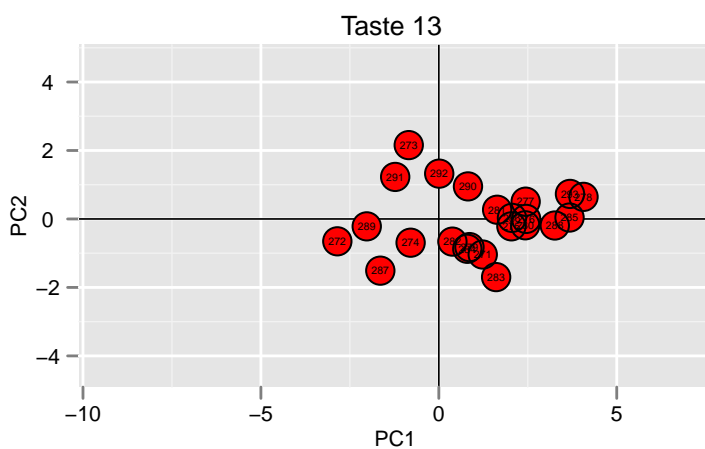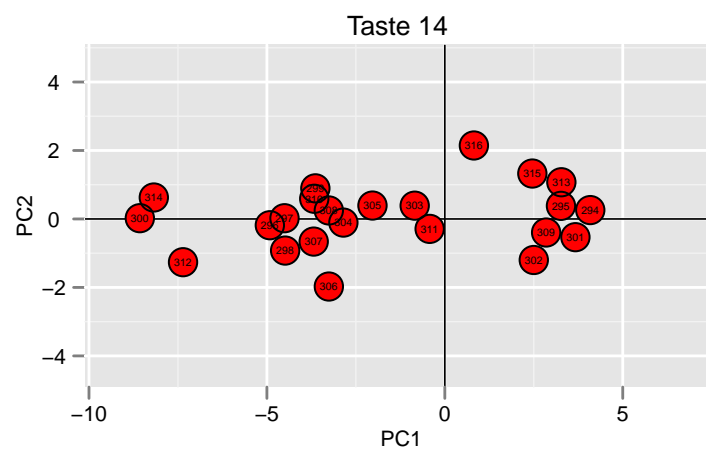

Supplement: Figure S2 — PCA biplot of the temporal product dynamics in liking. Analogously to the PCA biplot as in Figure S1, the temporal liking data are given in a biplot generated by a PCA on the complete data set of the Drinks Study. However, here the observations are given per product in every plot. These plots give insight in product modality. A unimodal product shows a large single cluster of observations (e.g. products 3 and 8). A bimodal product shows two clusters of observations (e.g. products 12 and 14). A multimodal product shows a large spread of observations (e.g. product 4). (PDF) [file pone.0093350.s002.pdf]

Liking scores for 4 clusters

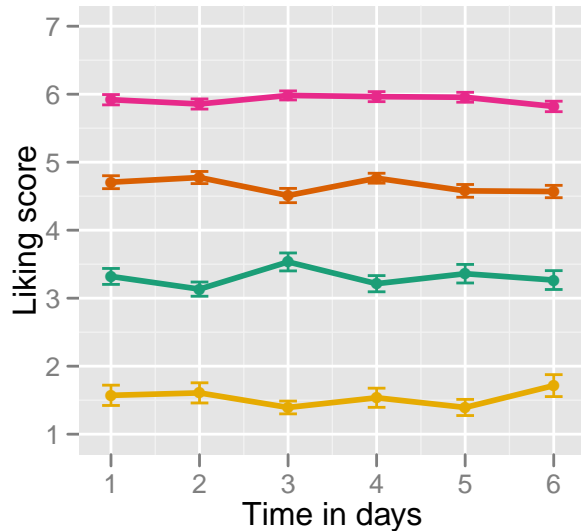

Liking scores for 5 clusters

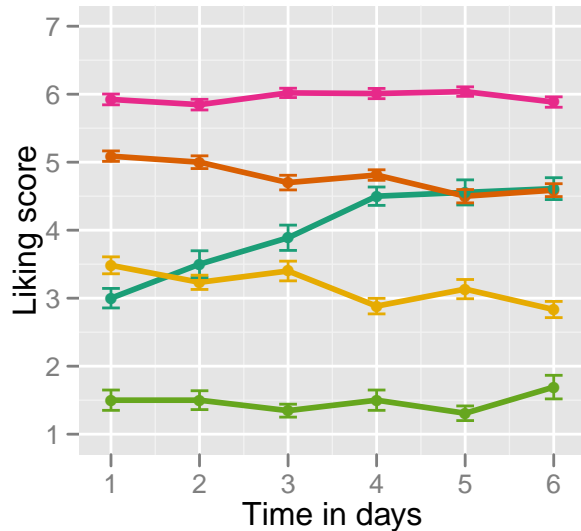

Liking scores for 6 clusters

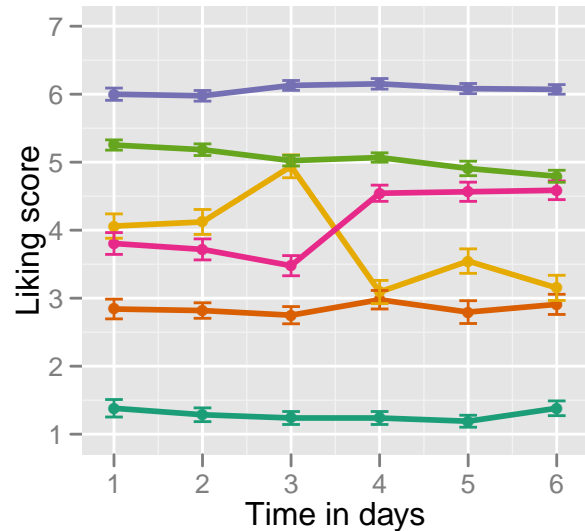

Supplement: Figure S3 — Liking during repeated exposure in the Drinks Study for 4,5 and 6 clusters. This figure shows the temporal dynamics of liking for 4,5 and 6 clusters in the Drinks Study. As can be seen the choice of 4 clusters will not show any information about the dynamics in time, whereas with five clusters these dynamics are visible. The choice of 6 clusters seems to overfit the data: a new fairly noisy cluster is introduced when the number of clusters increases from 5 to 6. (PDF) [file pone.0093350.s003.pdf]

Liking scores for 4 clusters

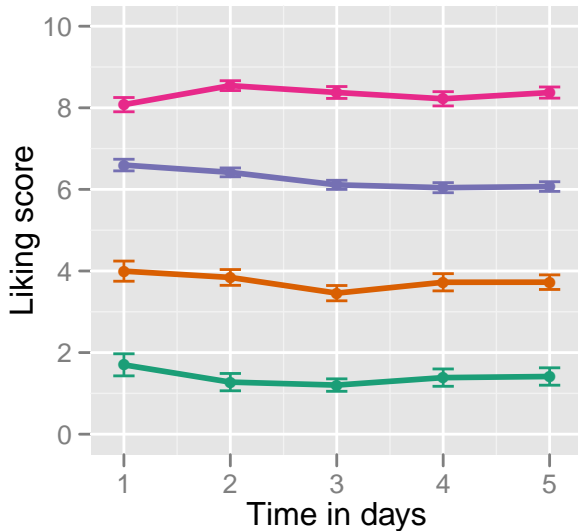

Liking scores for 5 clusters

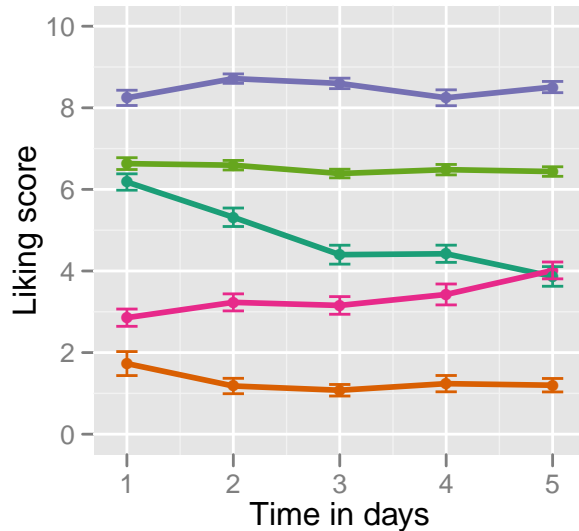

Liking scores for 6 clusters

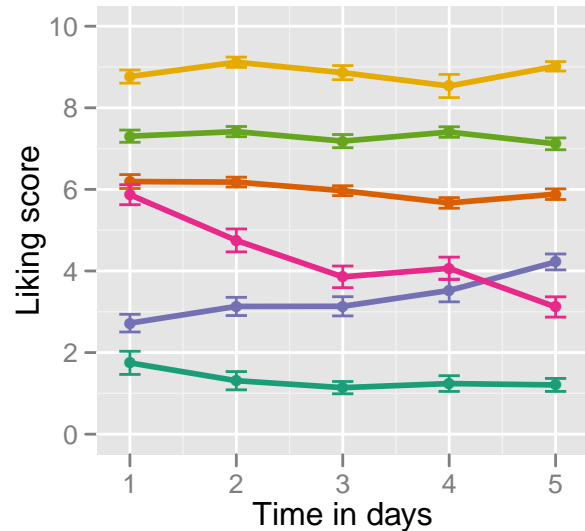

Supplement: Figure S4 — Liking during repeated exposure in the Snacks Study for 4,5 and 6 clusters. This figure shows the temporal dynamics of liking for 4,5 and 6 clusters in the Snacks Study. As can be seen the choice of 4 clusters will not show any information about the dynamics in time, whereas with five clusters these dynamics are visible. The choice of 6 clusters seems to overfit the data: the light green cluster with an intercept of ±6.5, (5 clusters), is split into two clusters (6 clusters) with intercepts of ±6 and ±7 respectively, showing very little information gain. (PDF) [file pone.0093350.s004.pdf]
