# Supplementary material for: Dealing with Consumer Differences in Liking during Repeated Exposure to Food; Typical Dynamics in Rating Behavior
Source: PLoS One. 2014 Mar 25;9(3):e93350. doi: 10.1371/journal.pone.0093350 (PMC3965558; doi:10.1371/journal.pone.0093350)
Supplement: Table S1 — Stimuli information from the Drinks Study. This table contains a more detailed description of all the products that were used in the Drinks Study. The products are subdivided in their associated product family. (DOCX) [file pone.0093350.s005.docx]

**S1 – Table containing the product types of the Drink Study**

| **Product Family** | **Composition** |
| --- | --- |
| Fortimel Extra | Milk protein concentrate, water, maltodextrin, vegetable oils, sucrose, acidity regulator (citric acid), emulsifier (soy lecithin), cocoa, flavoring (vanilla/apricot), tri-potassium citrate, choline chloride, calcium hydroxide, sodium L-ascorbate, potassium hydroxide, trisodium citrate, DL-α-tocopherol, ferrous lactate, nicotinamide, retinyl acetate, copper gluconate, manganese sulfate, zinc sulfate, sodium selenite, chromium chloride, D-calcium pantothenate, D-biotin, cholecalciferol, pyridoxine hydrochloride, pteroylmonoglutamic acid, thiamine hydrochloride, sodium fluoride, sodium molybdate, riboflavin, potassium iodide, phytomenadione.  (http://www.nutricia.de/productpdf/PN_Fortimel_Extra_308.pdf) |
| Forticare | Demineralised water, glucose syrup, sodium molybdate, milk protein isolate, sodium fluoride, trehalose, sucrose, vegetable oils, dietary fibres (oligofructose, inulin, cellulose, resistant starch), fish oil, whey protein concentrate (from milk), tri potassium citrate, flavour, sodium chloride, tri sodium citrate, colour (E150d), flavour, magnesium hydrogen phosphate, choline chloride, carotenoids (contains soy) (b-carotene, lutein, lycopene), sodium L-ascorbate, magnesium carbonate, potassium hydroxide, taurine, DL-a-tocopheryl acetate, L-carnitine, ferrous lactate, zinc sulphate, nicotinamide, retinyl aceteate, sodium selenite, manganese sulphate, copper gluconate, pyridoxine hydrochloride, calcium D-pantothenate, pteroylmonoglutamic acid, D-biotin, chromium chloride, cholecalciferol, cyanocobalamin, thiamin hydrochloride, sodium molybdate, sodium fluoride, riboflavin, potassium iodide, phytomenadione.  (<http://nutricia.co.uk/files/uploads/documents/FortiCare.pdf>) |
| DubbelFrisss | Apple/Peach: water, fruit juices from concentrated fruit juices, (apple 14%, peach 1.1%), citric acid, aroma, sugar, carbonic acid (< 0,1%). Total sugars/sodium (per 100gr): 8.6gr/0.001gr.  Apple/Berries: water, fruit juices from concentrated fruit juices, (apple 11%, blackcurrant 3%, aronia berry), citric acid, aroma, sugar, carbonic acid (< 0,1%). Total sugars/sodium (per 100 gr): 8.5gr/0.001gr.  Pineapple/Mango: water, fruit juices from concentrated fruit juices, (apple, pineapple 1%, mango 1%), citric acid, aroma, sugar, carbonic acid (< 0,1%). Total sugars/sodium (per 100gr): 8.6gr/0.001gr.  Orange/Tangerine: water, fruit juices from concentrated fruit juices, (orange 7%, apple, tangerine 2.4%), citric acid, aroma, sugar, carbonic acid (< 0,1%). Total sugars/sodium (per 100 gr): 8.6gr/0.001gr  ([http://www.dubbelfrisss.nl](http://www.dubbelfrisss.nl/)) |
| Optimel | Yogurt from skimmed milk, 5% fruit juices*, corn starch, calcium, sucralose. Proteins/Sugars/Sodium/Calcium (per 100gr): 3.1g/3.6g/0.04gr/120mg. Vitamins from the B complex (B2: 0,21mg; B6: 0,21mg; B12: 0,38 microgr).  * fruit juices, per product:  Raspberry: apple, 1% raspberry, aronia berry, lemon  Coconut: apple, lemon, 1% coconut  Lime: apple, 0.5% lemon, 0.5% lime  Orange-Cinnamon: apple, 1% orange, cinnamon  (<https://www.optimel.nl/>) |
| Saliva Orthana | Each 50 ml of aqueous solution contains: Mucin Gastric 1.75g, Xylitol 1.0g, Menthae Piperitae aetheroleum 2.5 mg, Spearmint Oil 2.5 mg, Methylis Parahydroxybonzoas 50 mg, Benzalkoni Chloridum 1 mg, -Disodium 25 mg, Sodium Fluoride 0.21 mg |
