# Supplementary material for: Dealing with Consumer Differences in Liking during Repeated Exposure to Food; Typical Dynamics in Rating Behavior
Source: PLoS One. 2014 Mar 25;9(3):e93350. doi: 10.1371/journal.pone.0093350 (PMC3965558; doi:10.1371/journal.pone.0093350)
Supplement: Table S2 — Determining the number of k-means clusters for both datasets. We used k-means to find homogenous groups of liking behaviors over time. The value of k (number of clusters) was chosen based on four different methods depicted in these tables. For BIC CL, BIC MAP and Gap Statistic the best values are in bold and indicated with a star. For WSS, the knee in the WSS plot determines the optimal number of clusters, which is an approximation based on visual inspection and indicated by X’s. As can be seen, there is little variation in the outcomes in both datasets; 4< = k < = 6. Visual inspection showed that the choice of 4 clusters did not provide any information about the temporal dynamics, while 6 clusters seemed to overfit the data (see Figures S3 and S4). (DOCX) [file pone.0093350.s006.docx]

**Drinks**

| **Clusters** | **WSS^1^** | **BIC MLE^2^** | **BIC MAP^3^** | **Gap Stat^4^** |
| --- | --- | --- | --- | --- |
| 2 |  | -807.4 | -6277.4 | 0.697 |
| 3 | X | -778.1 | -5969.0 | 0.877 |
| 4 | X | **-768.7*** | -5809.8 | 0.950 |
| 5 | X | -771.9 | -5821.8 | 0.954 |
| 6 |  | -777.3 | **-5771.6*** | **0.957*** |

**Snacks**

| **Clusters** | **WSS^1^** | **BIC MLE^2^** | **BIC MAP^3^** | **Gap Stat^4^** |
| --- | --- | --- | --- | --- |
| 2 | X | -601.0572 | -4340.03 | 1.024 |
| 3 | X | -579.0800 | -4121.86 | 1.218 |
| 4 | X | **-566.3401*** | **-4033.45*** | 1.343 |
| 5 |  | -567.3180 | -4044.47 | 1.358 |
| 6 |  | -570.5699 | -4042.18 | **1.362*** |

**^1^** Within sums of squares (WSS) curvature between values of k [28].

**^2^** Bayesian Information Criterion (BIC) based on maximum likelihood estimation (MLE) [25].

**^3^** Bayesian Information Criterion (BIC) based on maximum a posteriori (MAP) estimator with best model parameterization: VII (Spherical/Univariate, unconstrained)[27].

**^4^** The Gap Statistic [29].
